# Supplementary material for: SARS-CoV-2 Accessory Protein ORF8 Targets the Dimeric IgA Receptor pIgR
Source: Viruses. 2024 Jun 22;16(7):1008. doi: 10.3390/v16071008 (PMC11281603; doi:10.3390/v16071008)
Supplement: Supplementary file 1 [file viruses-16-01008-s001.zip › viruses-3047020-supplementary.pdf]

viruses

MDPI

Table S1. List of primers designed for mutagenesis of pIgR extracellular domain deletions.

**Primer Name**	**Primer sequence (5′->3′)**	**Tm (°C)**
Deletion Domain 1 (ΔD1)	cagctaagcttggtaccatgtttgatgtcagcctggaggt	56
Deletion Domain 1-2 (ΔD1-2)	cagcttaagcttggtaccatgctaaagcccgagcccgagct	64
Deletion Domain 1-3 (ΔD1-3)	caattaagcttatgccccgcagccccactgtggt	70
Deletion Domain 1-4 (ΔD1-4)	cagctaagcttggtaccatggaaggagaaccaaacctcaa	53
Deletion Domain 1-5 (ΔD1-5)	cagctaagcttggtaccatggttgaagagaggaaggcagc	57
**Primer Name**	**Primer sequence (3′->5′)**	**Tm (°C)**
Reverse primer (D5-1)	agaccagcgctctggagcttccaccttgtt	70

Supplementary Figures

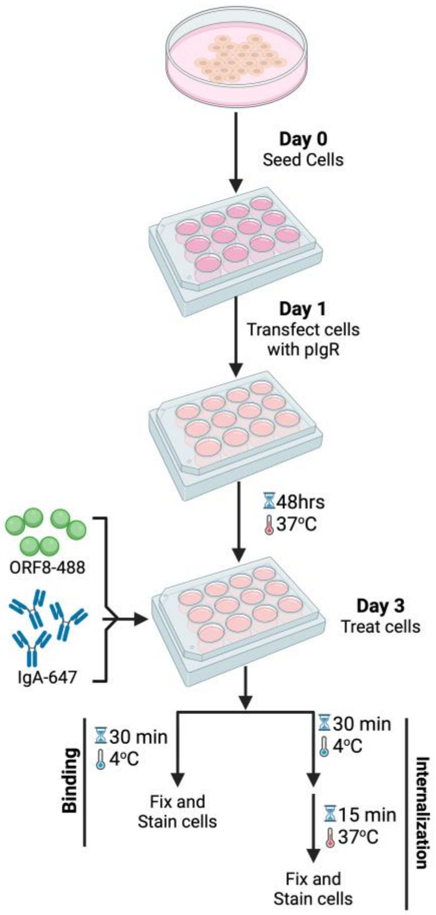

Figure S1. Workflow of ORF8 and IgA binding and internalization protocol for confocal microscopy and flow cytometry. Cells were seeded on day 0 and transfected on day 1 for 48 hours. On day 3, cells were treated according to the Methods and Materials with ORF8 and IgA, to observe either binding or internalization.

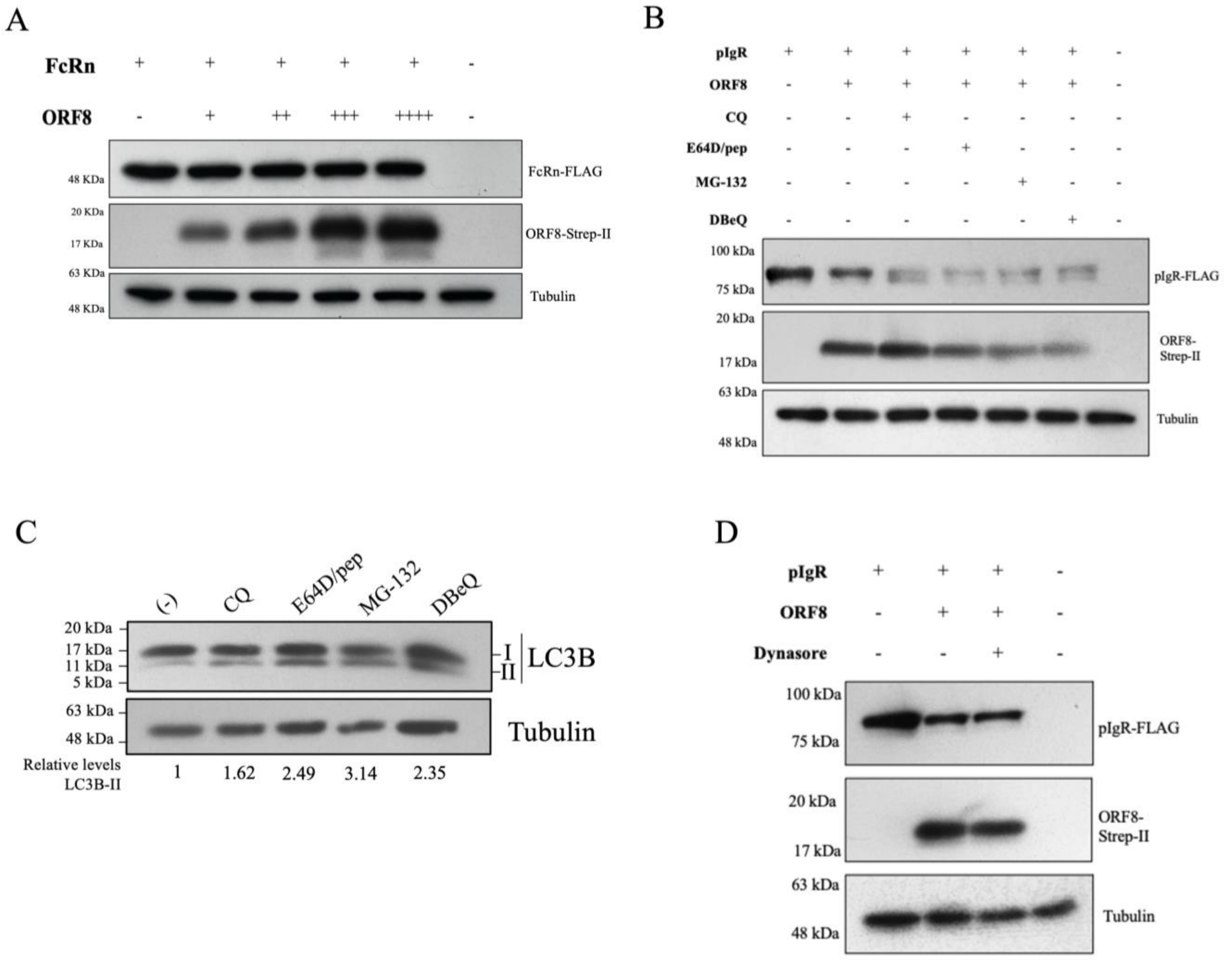

Figure S2. ORF8 downregulates pIgR specifically but not via degradation or endocytosis pathways . (**A**) HEK293T cells were co-transfected with 500 ng FcRn plasmid DNA and a titration of SARS-CoV-2 ORF8-Strep-II plasmid DNA (0 ng, 50 ng, 100 ng, 250 ng, 500 ng), and QCXIP DNA. (**B, C**) HEK293T cells were co-transfected with 500 ng pIgR, 250 ng QCXIP, and 250 ng SARS-CoV-2 ORF8. (**B-C**) Cells were treated with CQ (50 μM; 4 hours), E64d/pepstatin (10 μg/ml; 4 hours), MG-132 (10 μM; 6 hours), and DBeQ (15 μM; 6 hours). (**B**) Cells were treated with 16 μM Dynasore for 6 hours (**A-D**) Whole cell lysates were harvested and analyzed for pIgR-FLAG (**B, D**) or FcRn-FLAG (anti-FLAG) (**A**), ORF8-Strep-II (anti-Strep-II) (**A, B, D**), LC3B-I/II (anti-LC3B) (**C**) and tubulin (anti-tubulin) by Western Blot. (**C**) Intensities of protein bands were quantified using FIJI-ImageJ, and relative LC3B-II levels were normalized to tubulin. The value of the untreated control cells is arbitrarily set at 1.

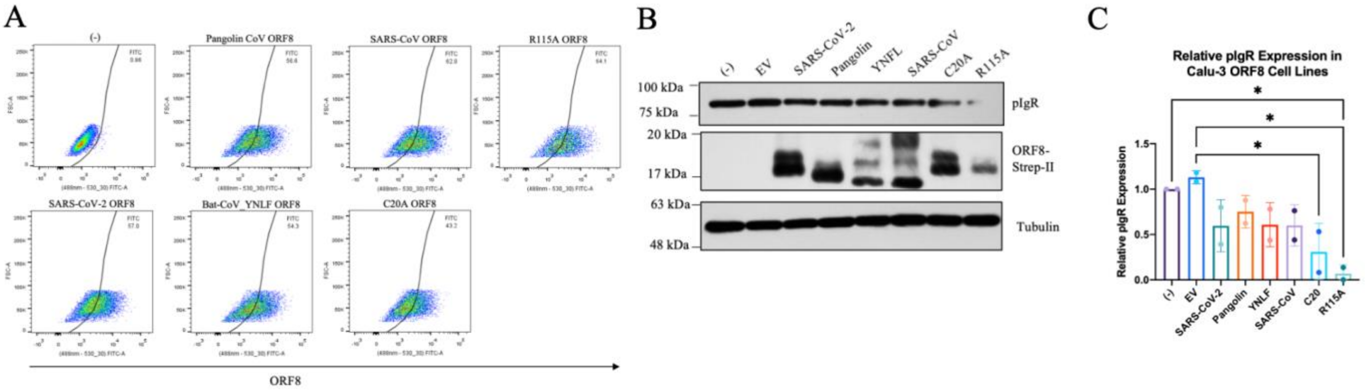

Figure S3. Effect of ORF8 on the endogenous pIgR in Calu-3 cells**.** (**A-C**) Calu-3 cell lines were generated to stably express SARS-CoV-2 ORF8-Strep-II and its mutants via transduction with lentiviral vectors carrying ORF8. (**A**) Calu-3 cells stained for ORF8-Strep-II (anti-Strep-II-FITC) and analyzed by flow cytometry. (**B-C**) Whole cell lysates were harvested and analyzed for pIgR (anti-pIgR), ORF8-Strep-II (anti-Strep-II), and tubulin (anti-tubulin) by Western blot. Protein expression was quantified using FIJI-Image J and PrismV9 (mean with SD; statistical significance measured via one-way Anova; * $p \leq 0.05$).

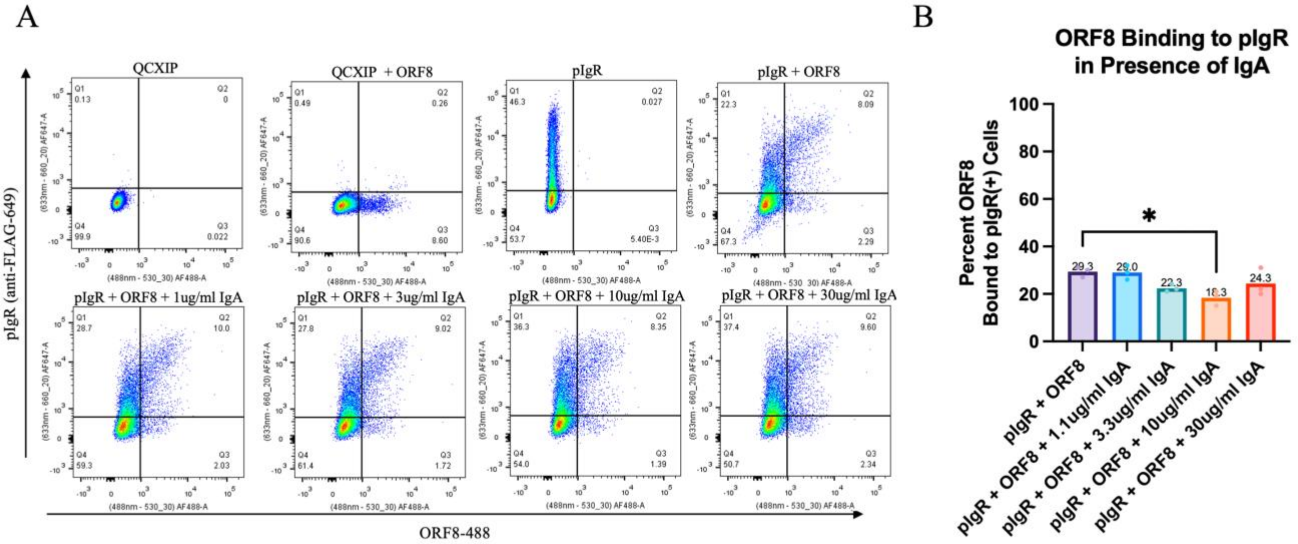

Figure S4. IgA competes with soluble ORF8 for pIgR binding. (**A-B**) HEK293T cells transfected with 250 ng pIgR and QCXIP and treated with 10 µg/ml ORF8-488 and a titration of IgA (1 µg/ml, 3 µg/ml, 10 µg/ml, 30 µg/ml) for 30 minutes on ice. ORF8 binding to pIgR-positive cells was analyzed with PrismV9 (mean with SD; statistical significance measured via one-way Anova; * $p \leq 0.05$).

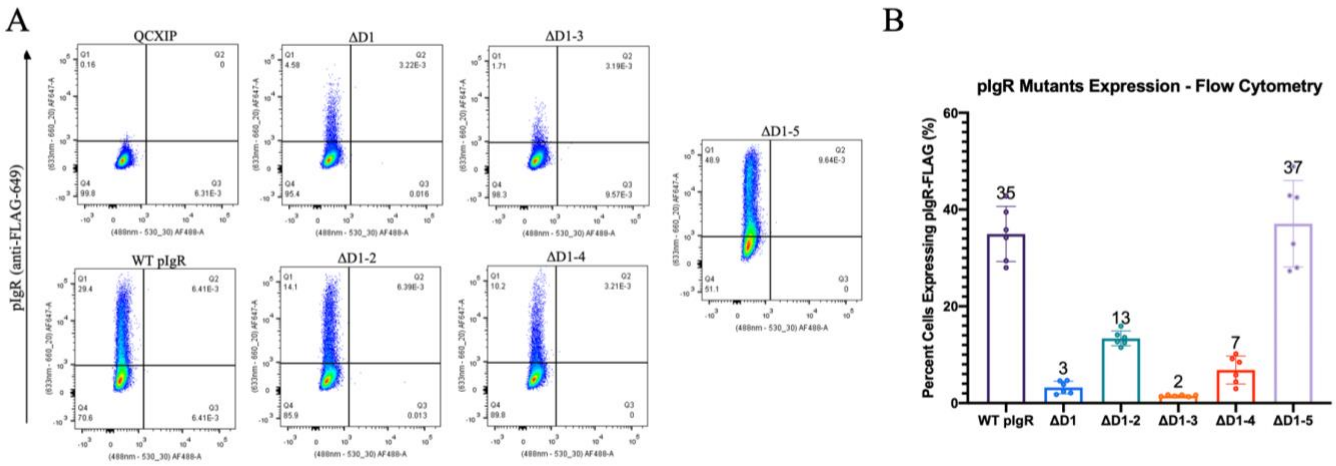

Figure S5. Expression of the pIgR mutants in HEK293T cells. **(A)** HEK293T cells were transfected with 250 ng pIgR or pIgR mutants and. Cells were stained with anti-FLAG (pIgR) and analyzed by flow cytometry. **(B)** Data were analyzed by PrismV9 (mean with SD).
